# Treatment Adherence in Dermatology During the COVID-19 Pandemic: A Review

**DOI:** 10.7759/cureus.34141

**Published:** 2023-01-24

**Authors:** Sarah G Bridgeman, Patrick O Perche, Steven R Feldman

**Affiliations:** 1 Dermatology, Wake Forest University School of Medicine, Winston-Salem, USA; 2 Dermatology, Pathology, and Social Sciences & Health Policy, Wake Forest University School of Medicine, Winston-Salem, USA

**Keywords:** covid-19, compliance, telemedicine, medication adherence, pandemic, dermatology, sars-cov-2

## Abstract

The coronavirus disease 2019 (COVID-19) pandemic raised many challenges for dermatology. Safety is a principal concern for many patients, particularly those on medications that affect the immune system. Understanding how the pandemic affects patients’ treatment adherence may be informative for counseling or other interventions to assure that treatment plans are not inappropriately interrupted, or for future pandemics. The purpose of this review was to investigate the extent to which the COVID-19 pandemic affected medication adherence in dermatology. A literature search of PubMed was performed using the search terms: adherence, compliance, dermatology, COVID-19, SARS-CoV-2 (severe acute respiratory syndrome coronavirus 2), and pandemic. Eleven primary research studies met the inclusion criteria and were included in the review. During the COVID-19 pandemic, non-adherence in dermatology patients was primarily linked to concern about the risk of COVID-19 infection with long-term use of immunomodulatory and immunosuppressive medications. In some cases, non-adherence was associated with feelings of depression, anxiety, or perceived stress. High adherence was attributed to regular and convenient communication between patients and dermatology providers through the effective use of telemedicine and electronic messaging. Frequent communication with providers to address patient concerns and provide continuity of care improved adherence. An integrated virtual approach to patient care facilitated this, particularly through the use of telemedicine. Implementation of routine virtual questionnaires was, to some extent, effective in replacing limited in-person patient-provider interactions during the pandemic. Finally, the added threat of the pandemic presented an additional mental health component to consider for patients, supporting the need for a multidisciplinary approach to patient care.

## Introduction and background

The coronavirus disease 2019 (COVID-19) pandemic raises many challenges for dermatology [1-3]. Safety is a principal concern for many patients, particularly those on medications that affect immune function [1,3-7]. Preventative discontinuation of biologic agents is not recommended; biologics potentially may limit the hyperinflammatory cascade of COVID-19. Nevertheless, patients may be fearful and not be fully adherent to anti-inflammatory treatments during the pandemic [1-3,5,8]. Even without the pandemic, non-adherence to dermatologic treatment is common and may be exacerbated by COVID-19 concerns [7,9,10]. Fear of COVID-19 infection and new financial burdens may also negatively impact health-seeking behaviors. Understanding how the pandemic affects patients’ treatment adherence may be informative for counseling or other interventions to assure that treatment plans are not inappropriately interrupted. The purpose of this review is to investigate the extent to which the COVID-19 pandemic has affected adherence in dermatology. 

## Review

Methods

**Figure 1:**
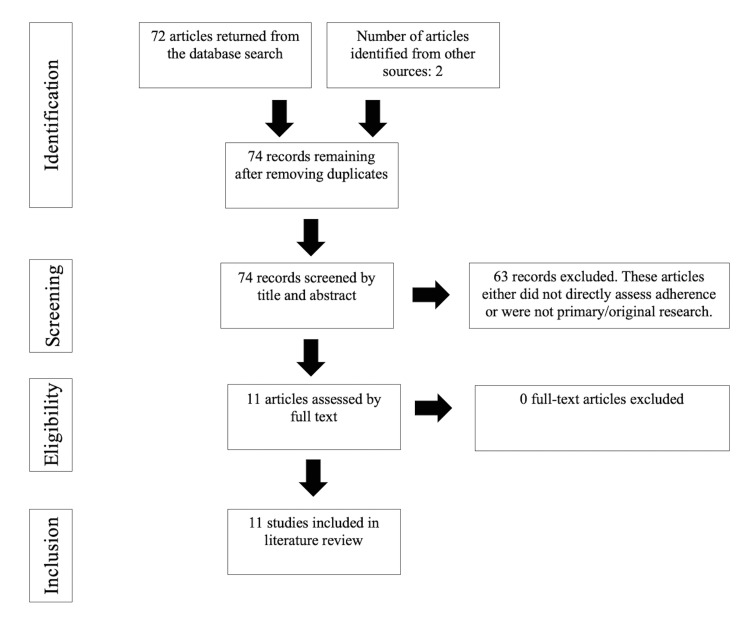
PRISMA Flow Diagram PRISMA: Preferred Reporting Items for Systematic Reviews and Meta-Analyses

A literature search was performed using PubMed and Cumulative Index to Nursing and Allied Health Literature (CINAHL) databases to identify articles relevant to the topic of adherence in dermatology during the COVID-19 pandemic. Search terms included: adherence, compliance, dermatology, COVID-19, SARS-CoV-2 (severe acute respiratory syndrome coronavirus 2), and pandemic. English articles published between January 1, 2020, and December 31, 2021, were included. The abstracts of all articles in the search return were read by the authors and those that were relevant to the topic were read in entirety and included as appropriate. Eleven relevant articles were included (Figure 1).

Eight articles addressed adherence in patients with psoriasis and three articles addressed adherence in patients with chronic urticaria (CU), basal cell carcinoma (BCC), and atopic dermatitis (AD), respectively (Table 1).

**Table 1:** Treatment adherence in dermatologic patients during the COVID-19 pandemic n: sample size; PSP: patient support program; COVID-19: coronavirus disease 2019

Date/Place	Authors	Study Type	n	Disease studied	Methods Used to Measure Adherence	Adherence Rate
2019-2020 Ankara, Turkey	Özdemir et al.[11]	Retrospective	1,525	Psoriasis	Adherence to follow-up; electronic follow-up data (routine submission system)	Greatest overall reduction in follow-up with biologic therapy (p < 0.001).
2020 Mainland China	Wang et al. [12]	Cross-sectional	926	Psoriasis	Treatment persistence; Online survey; Nonadherent if reduced drug dosage or completely discontinued use (Short Term: < 2 weeks; Long-term: > 2 weeks)	292 (31.5) adherent; 634 (68.5) non-adherent: 289 (31.2) short-term, 345 (37.3) long-term
2020 Toronto, Canada	Georgakopoulos and Yeung[13]	Retrospective	1,390	Psoriasis	Treatment persistence; PSP database review; Non-adherent if discontinued treatment	1383 (99.5) adherent 7 (0.5) non-adherent
2020 Thessaloniki, Greece	Vakirlis et al.[5]	Observational	237	Psoriasis	Treatment persistence; Phone interview; Responses verified using electronic pharmacy records for prescriptions/refills	181 (76.4) adherent, 56 (23.6) non-adherent
2020 Prague, Czech Republic	Rob et al.[14]	Qualitative	210	Psoriasis	Treatment persistence; Interview; Non-adherent if discontinued treatment	Biologics (n=177): complete adherence; Conventional immunosuppressants (n=47): 2 (4) discontinued treatment
2020 Istanbul, Turkey	Polat Ekinci et al.[15]	Qualitative	133	Psoriasis	Treatment persistence; Phone interview; Nonadherent if suspended treatment (Short term suspension: < 4 half-lives; Long term suspension: > than 4 half-lives)	52 (39) suspended biologic therapy: 33 (24.8) short-term, 19 (14.3) long-term. All but one restarted biologic therapy.
2020 Palermo, Italy	Tilotta et al.[3]	Observational	456	Psoriasis, Atopic Dermatitis, Hidradenitis Suppurativa	Treatment persistence; Regular check-in/interview; Non-adherent if discontinued treatment	454 (99.6) adherent 2 (0.4) non-adherent
2021 Istanbul, Turkey	Oguz Topal et al.[7]	Cross-sectional	342	Psoriasis	Treatment persistence; Survey; Oral therapy: Nonadherent if reduced dose or discontinued treatment for longer than 2 weeks; Injection therapy: Nonadherent if did not administer 1 or more doses	197 (57.6) adherent, 145 (42.4) non-adherent
2021 Istanbul, Turkey	Erdem et al.[16]	Observational	194	Chronic Urticaria	Treatment persistence; Interview; Non-adherent if discontinued treatment	138 (74.2) adherent, 48 (25.8) discontinued treatment
2020 Naples, Italy	Villani et al.[17]	Retrospective	37	Basal Cell Carcinoma	Treatment persistence; Tele-consultation; Non-adherent if discontinued treatment	Complete adherence to treatment
2021 Italy	Grieco et al.[18]	Qualitative	80	Atopic Dermatitis	Treatment persistence; Online survey; Non-adherent if discontinued treatment	73 (91.2) adherent, 7 (8.8) suspended treatment

Results

Psoriasis

A retrospective study conducted in Turkey between December 1, 2019, and August 31, 2020, used patient follow-up data to assess treatment adherence [11]. The number of patients who attended their follow-up appointments during the pre-COVID (PC) period (December 2019-February 2020), early-COVID (EC) period (March 2020-May 2020), and the late-COVID (LC) period (June 2020-August 2020) was measured. These data were used to compare adherence for patients on biologic therapy to those on non-biologic therapy. There were 1,525 patients with psoriasis included in the study, of which 1,084 were treated during the PC period. During the EC period, 8.5% and 18.1% of PC patients using biologics and non-biologic therapy, respectively, attended their follow-up appointments (Table 1). Similar findings were observed in the LC period, where 10.9% and 22.7% of patients were followed up in the biologic and non-biologic groups, respectively. The study also assessed rates of patients initiating biologics during the pandemic, and there was an increase in biologic initiation during the EC and LC periods compared to the PC period. Authors speculate that this increase was secondary to disease deterioration from poor follow-up and non-adherence to treatment. They state that poor adherence and follow-up, especially in patients on biologics, may have been due to limited access to healthcare or concern about susceptibility to COVID-19. At the onset of the pandemic, they state many dermatologists were not certain how to advise patients with psoriasis regarding biologic use in the setting of COVID-19.

An online questionnaire assessed patients with psoriasis in China on their adherence rates from February 2020 until March 2020 [12]. The questionnaire was posted on dermatology media platforms, and patients were introduced to these platforms by their physicians. Patients who participated in the study were on a range of treatments: biologics, systemics, and topicals. Patients were categorized as short-term non-adherent (discontinuation of medication use for less than two weeks) and long-term non-adherent (discontinuation of medication use for greater than two weeks). Only 31.5% of patients were adherent to their medications (Table 1). The prevalence of non-adherence was greater for patients on systemic (p = 0.003) or topical (p < 0.001) therapy compared to those on biologics. Non-adherence was associated with worsening and deterioration of patients’ psoriasis (adjusted odds ratio (AOR): 2.83 to 5.25, non-adherence < 2 weeks, non-adherence > 2 weeks, respectively). There was also an association between non-adherence and feelings of anxiety (AORs: 1.42 to 1.57), depression (AORs: 1.78 for both), and perceived stress (AORs: 1.86 to 1.57). The authors note that non-adherence rates in this study were higher compared to previous studies in China, and these findings suggest that the pandemic may have had a negative impact on adherence behavior in psoriasis patients.

Adherence in Canadian patients with psoriasis on biologics was assessed from February 1, 2020, until April 15, 2020 [13]. Adherence data were obtained through the patient support program (PSP) case managers database. Nearly 100% of Canadian psoriasis patients are enrolled in this program, and a patient’s PSP is normally notified in the case of patient-driven treatment discontinuation. Adherence was remarkably high with 99.5% of patients remaining adherent (Table 1). However, when non-adherence did occur, it was due to concern for increased susceptibility to COVID-19. No patients discontinued treatment due to active COVID-19 infection. The authors attribute a high medication adherence rate to their use of telemedicine visits, which allowed them to address patient concerns about their treatment during the pandemic.

An observational study in Greece interviewed patients with psoriasis over the phone from March to April 2020 regarding medication adherence during the pandemic [5]. Responses were verified using electronic pharmacy records. Patients with psoriasis on systemic medications and biologics were included in the study. The adherence rate was 76.4% during the pandemic (Table 1). According to the interviews, discontinuation of medication was exclusively due to concern about susceptibility to COVID-19 infection. Adherence rates were not statistically significantly different based on patients’ medication, age, or the presence of specific comorbidities. However, the authors noted that patients with greater than three comorbidities were more than six times more likely to be non-adherent (p = 0.03). Many patients participating in this study (57%) had comorbidities putting them at higher risk for infection with COVID-19. The authors note the adherence rate of 76.4% is largely attributed to maintaining patient access to treatment despite the limitations of the pandemic. They report success with telecounseling, electronic prescription services, and effective communication via text and email.

A study conducted in the Czech Republic assessed adherence by interviewing psoriasis patients during their national lockdown period of the COVID-19 pandemic (16 March to 24 April 2020) [14]. All psoriasis patients who were scheduled for a visit with their dermatologist during this period were enrolled in the study. Of 210 participants in the study, 55.7% were on biologic therapy, 22.4% were on conventional immunosuppressive therapy, and the remaining 21.9% of patients were on topical therapy. No patients on biologics discontinued treatment while 4.3% of patients on conventional immunosuppressants discontinued treatment, all due to concern for susceptibility to COVID-19 (Table 1). Patients were asked to respond to the statement “I feel an increased risk of infection from COVID-19 because of the type of treatment for my psoriasis.” Patients could respond with strongly disagree, disagree, don’t know, agree, or strongly agree. There was a statistically significantly greater concern for safety in patients on biologics compared to those on conventional immunosuppressive medications (p < 0.01) and topicals (p < 0.00001). Patients also filled out the anxiety portion of the Hospital Anxiety and Depression Scale (HADS), HADS-A. Patients on biologic therapy had a higher prevalence of anxiety and a higher average HADS-A score compared to those on topical therapy (p < 0.01 and p<0.04, respectively). Overall, patients on biologics remained adherent despite their greater prevalence of anxiety, higher average HADS-A scores, and greater concern for susceptibility to COVID-19.

From June 20, 2020, to June 28, 2020, Turkish patients with psoriasis from a dedicated psoriasis clinic were recruited and interviewed over the phone by a dermatologist, assessing their attitudes and perceptions about COVID-19 as well as their adherence to psoriasis therapy and their current psoriasis disease severity [15]. All patients on biologics during the pandemic were eligible for inclusion in the study. Prior to the interviews, patients were contacted at the onset of the pandemic (March 11, 2020) and informed that they should continue biologic therapy unless they experienced symptoms consistent with COVID-19 infection or tested positive for COVID-19. Patients were also informed of how to safely obtain their medications. If treatment was suspended during the pandemic, patients were classified into “short-term suspended” and “long-term suspended” groups based on whether the duration of suspension was less than or greater than four half-lives of the biologic agent. From the interviews, 39.1% of patients suspended biologic therapy: 24.8% short-term, 14.3% long-term (Table 1). The most common reasons for non-adherence included feelings of fear, anxiety, or worry regarding their susceptibility to COVID-19 infection while on biologic therapy. However, all patients who remained adherent to treatment also reported these feelings about their immunosuppression status during the COVID-19 pandemic. Although non-adherence in this study was primarily short-term, overall adherence was poor despite early efforts for patient education. Nonetheless, of all patients who suspended treatment, all but one restarted their biologic therapy after further discussion with their dermatologist. Authors attribute this improvement to strong relationships and consistent communication with patients. 

In 2020, an observational study was conducted to assess adherence during the lockdown phase of the COVID-19 pandemic in Italy [3]. Patients were contacted regularly by their dermatology provider to check in and inquire about adherence. Patients were labeled non-adherent if they reported discontinuation of their treatment. Of 456 patients, only two discontinued treatment (Table 1). Both patients who discontinued treatment reported that this was due to fear of infection with COVID-19; however, both re-initiated therapy after a two-month interruption. Twenty-five patients increased the length of their dosing interval by a range of 14-37 days but did not completely discontinue treatment. This was noted only in the early lockdown period and attributed to patient misinformation. The authors attribute high overall adherence to their use of telemedicine, regular check-in with patients, and patient accessibility to their pharmacy.

A cross-sectional study was conducted during the pandemic (May 2021-August 2021) in eight Turkish dermatology clinics using a detailed questionnaire to assess adherence [7]. The questionnaire also included the Psoriasis Area Severity Index (PASI), Dermatology Life Quality Index (DLQI), and HADS assessment. Visual Analog Scale (VAS) scores were recorded for patients who discontinued treatment or experienced an increase in psoriasis lesions. All patients were either on oral therapy (23.1%) or biologics (76.9%). Adherence was defined based on the type of systemic therapy. Patients on oral therapy were classified as non-adherent if they reduced medication dosage or discontinued treatment for longer than two weeks. Patients on injection treatment were classified as non-adherent if they did not administer one or more doses of the medication. Based on this, 57.6% of patients were adherent to their treatment and 42.4% were non-adherent (Table 1). There was no statistically significant relationship between adherence and the type of treatment (injection therapy vs. oral therapy), age, or the presence of comorbidities. Patients were reportedly non-adherent largely due to inability to access the hospital/medication (26.5%), inability to reach their provider (7.3%), concern about susceptibility to COVID-19 (16.3%), or active COVID-19 infection (3.8%). Less frequently reported reasons included adverse events (1.7%), forgetfulness (0.8%), financial problems (0.5%), improvement in psoriatic lesions (0.5%), and secondary ineffectiveness (0.2%). Other patients (13.7%) discontinued treatment based on provider recommendation. Non-adherence was associated with a longer duration of drug treatment prior to the pandemic (p < 0.001); however, there was no statistically significant relationship between disease duration and adherence. Non-adherence was also associated with increased psoriasis severity (higher mean VAS scores, PASI ≥ 10, DLQI ≥ 10; p < 0.001). There was no statistically significant relationship between depression or anxiety and adherence based on HADS scores (p > 0.05).

Chronic Urticaria 

An observational Turkish study evaluated patient adherence through patient interviews in the allergy units of two dermatology departments [16]. Patient interviews took place during the COVID-19 pandemic, after June 1, 2020. Patients were on various medications for CU including systemic antihistamine (72.2%), omalizumab (17.5%), antihistamine-montelukast (2.6%), and/or systemic corticosteroid (8.6%). Patients were divided into two groups: aggravated and non-aggravated. This was based on Urticaria Activity Scores (UAS7) from before the onset of the pandemic (before March 11, 2020) and during the pandemic (after June 1, 2020). Patients in the “aggravated” group had a UAS7 score > 6 prior to the pandemic, > 7 during the pandemic, or a minimum increase of 7 points between these two periods. Of 186 patients who began treatment prior to March 1, 2020, 74.2% remained adherent to their medications (77.9% on antihistamine therapy, 77.8% on systemic corticosteroids, 55.9% on omalizumab, antihistamine-montelukast not reported) (Table 1). Adherence was not statistically different between aggravated and non-aggravated groups. Patient reasoning for treatment discontinuation was not mentioned, although it is highlighted that a large percentage (89.2%) of patients reported an inability to contact a dermatologist during the pandemic; 45.4% attributed this to outpatient clinic closures and 29.9% to fear of COVID-19. The remaining patients (17.5%) cited other causes. 

Basal Cell Carcinoma

A retrospective study in Naples, Italy, assessed adherence in patients with advanced BCC being treated with sonic hedgehog inhibitors during the COVID-19 pandemic in 2019 [17]. Nine patients were on sonidegib and 28 were on vismodegib. Teleconsultations were utilized to organize therapeutic management and avoid treatment discontinuation during the pandemic. Treatment was discontinued by the provider in one of the sonidegib patients and two of the vismodegib patients, all due to the presence of severe comorbidities. Otherwise, patients were completely adherent to treatment (Table 1). Patients reported that this was largely due to concern for the effect of treatment interruption on their condition, despite adverse events, and the authors note the utilization of teleconsultation as a favorable strategy. 

Atopic Dermatitis

A qualitative study followed adult AD patients treated with dupilumab to assess treatment adherence in Italy during the lockdown period of the pandemic (February 3, 2020, to May 29, 2020) [18]. Patients were consulted via routine teleconsultation and asked to participate in this study and take an online survey. Survey questions addressed demographic information, risk factors for COVID-19 infection, and management of dupilumab therapy. There were 80 patients who enrolled in the study, and 59 who completed the surveys (73.8%). Regardless of survey completion, all 80 patients received monthly follow-up phone calls to collect patient-reported outcomes regarding their AD severity, as well as assess adherence to treatment. Adherence was measured based on treatment persistence during the lockdown period, and patients who discontinued treatment were classified as non-adherent. Of the 80 patients in the study, 73 (91.2%) were adherent to treatment based on follow-up phone calls (Table 1). The remaining seven patients who suspended treatment did so due to personal concern (5), difficulty accessing their medication (1), and physician recommendation (1). The authors suggest that the survey intervention itself, along with the follow-up phone calls, may have attributed to high overall adherence, serving as an additional means of communication during a time of limited social interaction.

Discussion

Dermatology research evaluating adherence during the COVID-19 pandemic is largely focused on inflammatory and immune-mediated conditions (psoriasis, CU, BCC, and AD). Non-adherence in these patients has been primarily linked to concern about the risk of COVID-19 infection with long-term use of immunomodulatory and immunosuppressive medications [3,5,7,14-16,18]. Notably, many patients who participated in these studies had comorbidities that put them at an increased risk for infection with COVID-19 [5,7,15,18]. While the presence of these conditions may not directly impact the treatment of dermatologic problems, this may contribute to patient fear about COVID-19 susceptibility.

One of the ways to manage this fear is through patient education [3,15]. In one study, non-adherence still occurred despite educating patients early on that they should continue biologic therapy during the pandemic. However, adherence largely improved after further discussion with a dermatology provider [15]. Other studies made similar observations: frequent communication with providers to address patient concerns and provide more continuity in patient education may improve adherence [3,5,13].

Unfortunately, COVID-19 introduces new communication barriers, in part by limiting access to healthcare resources during isolation. These limitations contributed to non-adherence [7,16,18]. Technology is of growing influence in helping providers adapt to these pandemic-related changes. Telemedicine and electronic messaging may improve adherence, facilitating regular, convenient communication between patients and dermatology providers [3,5,13]. Since contact with patients improves adherence, the surveys done in survey studies may improve adherence [18]. Implementing virtual questionnaires routinely may, to some extent, replace limited in-person patient-provider interactions during the pandemic.

COVID-19 can affect mental health which in turn can impact adherence behavior. One study found no statistically significant relationship between adherence and depression or anxiety [7]. Another study reported a higher prevalence of anxiety for patients on biologics compared to topicals; however, this was not associated with non-adherence [14]. Conversely, two studies found an association between non-adherence and feelings of depression, anxiety, or perceived stress [12,15]. Depression and increased stress are associated with poor adherence and are linked to the worsening of chronic dermatologic diseases [1,2,6,19,20]. Thus, the added threat of the pandemic may present an additional mental health component to consider for dermatology patients, supporting a multidisciplinary approach to patient care.

The methods used to measure adherence in these studies were largely subjective, in the form of questionnaires and interviews. Electronic surveys are a helpful way to encourage adherence and maintain communication with patients, but this form of data collection may exclude less accessible groups such as pediatric or geriatric populations. Additionally, these methods to measure adherence are subject to recall bias. In contrast, objective data such as electronic follow-up records, pharmaceutical records, and case management databases are helpful for assessing adherence, but this information excludes the patient's perspective. In the setting of a global pandemic, monitoring adherence should ideally include a combination of these metrics with an emphasis on maintaining contact with patients via telehealth or electronic messaging. This communication is a key component in equipping patients with the tools necessary to continue treatment during times of isolation with poor access to treatment facilities. Regarding differences in adherence to medications that were self-administered vs office-administered, most of the studies examined did not assess this. However, one study did assess this, and there was no statistically significant difference between adherence to injection vs oral therapy [7]. Nonetheless, multiple studies emphasized the importance of working with pharmacies to ensure that patients could access medications during the lockdown periods of the pandemic [3,5].

## Conclusions

In conclusion, the COVID-19 pandemic introduced new barriers to medication adherence in the field of dermatology. Non-adherence in dermatology patients was primarily linked to concern about risk of COVID-19 infection with long-term use of immunomodulatory and immunosuppressive medications. In some cases, non-adherence was associated with feelings of depression, anxiety, or perceived stress. High adherence was attributed to regular and convenient communication between patients and dermatology providers though the effective use of telemedicine and electronic messaging. Frequent communication with providers to address patient concerns and provide continuity of care improved adherence. An integrated virtual approach to patient care facilitated this, particularly through the use of telemedicine. Implementation of routine virtual questionnaires was, to some extent, effective in replacing limited in-person patient-provider interactions during the pandemic. Finally, the added threat of the pandemic presented an additional mental health component to consider for patients, supporting the need for a multidisciplinary approach to patient care. Overall, improved implementation of virtually-integrated and multidisciplinary treatment plans were helpful in maintaining and improving adherence in the setting of COVID-19. These lessons and experiences with patient care in the setting of the COVID-19 pandemic may be highly informative if and when future pandemics arise. 
